# A trans-disciplinary approach to the evaluation of social determinants of health in a hispanic population

**DOI:** 10.1186/1471-2458-12-769

**Published:** 2012-09-11

**Authors:** Michael F Dulin, Hazel Tapp, Heather A Smith, Brisa Urquieta de Hernandez, Maren J Coffman, Tom Ludden, Janni Sorensen, Owen J Furuseth

**Affiliations:** 1 Department of Family Medicine, Carolinas HealthCare System, 2001 Vail Avenue, Charlotte, NC, 28207, USA; 2 Department of Geography and Earth Sciences, University of North Carolina at Charlotte, 9201 University City Blvd, Charlotte, NC, 28223, USA; 3 School of Nursing University of North Carolina at Charlotte, 9201 University City Blvd, Charlotte, NC, 28223, USA; 4 Metropolitan Studies and Extended Academic Programs University of North Carolina at Charlotte, 9201 University City Blvd, Charlotte, NC, 28223, USA

**Keywords:** Social determinants of health, Community-based participatory research, Geospatial models, Hispanic

## Abstract

**Background:**

Individual and community health are adversely impacted by disparities in health outcomes among disadvantaged and vulnerable populations. Understanding the underlying causes for variations in health outcomes is an essential step towards developing effective interventions to ameliorate inequalities and subsequently improve overall community health. Working at the neighborhood scale, this study examines multiple social determinates that can cause health disparities including low neighborhood wealth, weak social networks, inadequate public infrastructure, the presence of hazardous materials in or near a neighborhood, and the lack of access to primary care services. The goal of this research is to develop innovative and replicable strategies to improve community health in disadvantaged communities such as newly arrived Hispanic immigrants.

**Methods/design:**

This project is taking place within a primary care practice-based research network (PBRN) using key principles of community-based participatory research (CBPR). Associations between social determinants and rates of hospitalizations, emergency department (ED) use, and ED use for primary care treatable or preventable conditions are being examined. Geospatial models are in development using both hospital and community level data to identify local areas where interventions to improve disparities would have the greatest impact. The developed associations between social determinants and health outcomes as well as the geospatial models will be validated using community surveys and qualitative methods. A rapidly growing and underserved Hispanic immigrant population will be the target of an intervention informed by the research process to impact utilization of primary care services and designed, deployed, and evaluated using the geospatial tools and qualitative research findings. The purpose of this intervention will be to reduce health disparities by improving access to, and utilization of, primary care and preventative services.

**Discussion:**

The results of this study will demonstrate the importance of several novel approaches to ameliorating health disparities, including the use of CBPR, the effectiveness of community-based interventions to influence health outcomes by leveraging social networks, and the importance of primary care access in ameliorating health disparities.

## Background

Progress in the efforts to reduce health disparities for vulnerable populations using traditional research methods can be characterized as modest and advancing slowly [1,2]. One potential solution for more rapidly improving health outcomes and reducing health disparities is the use of community-based participatory research (CBPR) to identify and address the relationship between social determinants (such as income, housing density, access to food and parks, exposure to pollution, and social networks) and health outcomes and translate those findings into community based interventions [3,4]. Use of the CBPR framework allows for the rapid translation of research evidence into real-world settings and marshals collaborative social action to support sustainable change. These types of CBPR programs are designed to develop specific community-based interventions that impact health policy and effects [4,5]. Although CBPR has been offered as a means of promoting community relationships and providing a framework for community interventions, there are still only a handful of published studies that demonstrate the effectiveness of CBPR in directly influencing health outcomes, indicating a clear need for additional studies [6-10].

While this project broadly examines the social determinants of health, the intervention design and implementation are focused upon the Hispanic population. This population has been the main focus of the research within our network [11-15], and partnerships with Hispanic leaders and organizations are already in place to facilitate the completion of this project. Charlotte, North Carolina provides an ideal setting in which to identify new ways to mitigate barriers and improve health outcomes for disadvantaged Hispanic residents. Charlotte is the center of the fourth fastest growing Hispanic Hypergrowth metro in the U.S. Between 1980-2000, Hispanic in-migration led to a 932 percent increase in the Latino population. By 2010, 13.1% of Charlotte’s population was Hispanic [16-19]. As a group, Charlotte’s Hispanics are less educated and poorer than any other racial or ethnic group. An estimated 65-70% of all Hispanic adults lack health insurance [20]. Indeed, the most recent data indicate that about 27% of Charlotte’s Hispanic population lives at or below the poverty level. From a health status perspective, Charlotte-Mecklenburg’s Hispanic population present disparities in the following diseases and conditions: immunization rates; access to prenatal care; HIV infection and death; death from motor vehicle accidents and homicide; teen pregnancy; sexually transmitted infections; overweight children and adults [21]. Many institutional and immigration barriers prevent the Hispanic population from accessing medical care, negatively affecting overall community health [22-26]. Not surprisingly, North Carolina’s Hispanic population has the lowest rate of routine medical care for any ethnic group in the state, 41.1% versus 7.3% for African-Americans and 13.7% for Whites [27].

Immigration scholars have labeled Charlotte as a pre-emerging gateway. This construct describes urban areas with little experience receiving large numbers of immigrants in a short period of time that also lack public service systems that are oriented to meet the needs of low wealth, non-English speaking clients [17]. Consequently, the rapid influx of new Hispanic residents has challenged health care providers and facilities. Nonetheless, without the prejudice against newcomers that is widespread in traditional immigrant gateways, Charlotte has a unique opportunity to create constructive relationships between medical providers and the Hispanic community to proactively and positively impact community health; improve cultural understanding; and break down barriers between community members and health providers [17].

Improving access to primary care and preventive health services is a powerful strategy for reducing health disparities for underserved and disadvantaged populations [7,28-31]. The evidence for this correlation is based upon large-scale retrospective studies looking at the healthcare workforce where increases in the number of primary care physicians within the healthcare delivery system are associated with improvements in health disparities [31-34]. These findings have hold true for the Hispanic immigrant population [35]. However, there are no published studies that have prospectively shown that increased access to and utilization of primary care services at a local level can impact health outcomes or ameliorate disparities. Indeed, despite the call for increased access to primary care services that has accompanied healthcare reform, there is evidence of a potential negative association [36].

Measuring data and examining health inequalities at a neighborhood scale has been greatly enhanced by new spatial modeling enhancements. In particular, Geographic Information Systems (GIS) permit the mapping of variables within a neighborhood or sub-neighborhood geography in order to examine the spatial relationships between health predictors and outcomes [37-39]. In the past, mapping tools were primarily used to examine the patterns of disease transmission [40-42]. But, these tools can also be used effectively to evaluate patterns of healthcare access and to define community service areas [43,44]. For example, geographic retrofitting methods define the service areas of medical facilities allowing for analysis of service delivery and intervention design [44]. This technique will be used as a means to evaluate primary care service delivery areas which can be followed over time to evaluate changes in patterns of healthcare access.

The PBRN model provides an ideal setting for examining interventions that improve access to primary care services. The PBRN is designed to help clinicians better understand and overcome obstacles facing primary care providers as they seek to improve community health. Integrating community-participation within a network of providers has been suggested as a way to bridge the gap between the medical system and the community [11,45].

This research study will use a CBPR within a PBRN to achieve four tasks: 1) to examine the relationship between social determinants and health outcomes for the Charlotte-Mecklenburg community; 2) to use community and hospital data to develop geospatial models that identify areas within our community that would most benefit from increased access to primary care; 3) to validate these geospatial models using patient surveys and qualitative analysis; and 4) to build, implement and evaluate a community-based intervention to improve access to primary care for the Hispanic immigrant population.

The PBRN managing this study, The Mecklenburg Area Partnership for Primary-care Research (MAPPR), has previously employed elements of CBPR and geospatial modeling to create preliminary models of community-wide healthcare utilization [13,14]. In this research, these models will be enhanced and validated and then deployed to build and direct interventions to improve access to care. Although geospatial models have significant potential to monitor changes in patterns of healthcare utilization and health outcomes, currently there are no studies that have used geospatial models in combination with qualitative analysis to develop, implement, monitor, and evaluate community interventions and study social determinants of health.

### Aims

1. A centralized database will be created with the goal of examining correlations between specific social determinants and health outcomes at both the community wide and neighborhood levels. The database will bring together information from the healthcare system with community-centered information, including demographics; socioeconomic status; social networks; and access to healthy foods, preventative health services, and transportation.

2. Geospatial models that target neighborhoods that would most benefit from increased access to primary care and preventative services will be created through modeling the data from Aim 1 to identify associations between specific social determinants and health outcomes for the targeted neighborhoods.

3. The geospatial models will be validated using qualitative methods and surveys.

4. An intervention to positively change the social networks of low-income Hispanic populations within the target neighborhood will be designed, implemented, and evaluated using CBPR techniques, in order to impact the Hispanic community’s utilization of primary care services.

### Methods/design

This study received ethics approval from the Institutional Review Board of Carolinas HealthCare System.

### Setting

The research network, MAPPR, includes the hospitals, urgent care centers, and outpatient clinics within Carolinas Healthcare System (CHS), community organizations, area safety-net clinics, the county health department; UNC Charlotte’s Metropolitan Studies Division and Department of Geography and Earth Sciences; UNC Charlotte’s College of Health and Human Services; and the Charlotte-Mecklenburg Schools. The network’s community clinics care for over 85% of the city’s uninsured patients and managed over 225,000 visits in 2009. These clinics, in addition to the county health department and five area hospitals, serve the majority of the city’s disadvantaged patients and all are part of a large, vertically integrated healthcare system (CHS) that shares a common informatics system.

### The community-based participatory research (CBPR) framework

The key principles of CBPR are utilized to evaluate social determinants of health and develop an applicable and culturally appropriate intervention with collaborative partnerships through a community advisory board (CAB). Specifically, two levels of CBPR are being utilized. First, the CAB is in place and will oversee all aspects of the study. This board includes community members, researchers, providers, and other key stakeholders from the community. These Hispanic leaders and service providers have roles where they can use the findings from this project to impact policy and/or find additional resources to make changes in community and neighborhood infrastructure. Finally, this group will have access to the study data, and will plan and direct the research project with the assistance of the research team. Second, focus groups made up of key constituencies will inform the project management. Group membership is comprised of service providers and Hispanic residents. These groups of 6 to 8 participants will be asked to assist in directing the research project and will be asked to address specific questions generated by the CAB. For example, they will be asked to give input on the validity of geospatial models and to assist in the development of community-based interventions.

### Database development

At the core of this research is the development of a centralized database for measuring and evaluating community-level data. This database will examine correlations between specific social determinants and health outcomes at both the city/county scale, as well as, neighborhood levels. The database will be compiled with primary and secondary data from the Carolinas Healthcare Systems billing and clinical databases (clinical and demographic data from 95 primary care clinics and 32 regional facilities including CHS hospitals, emergency departments, urgent care centers specialty clinics); Medicaid data from Community Care Partners of Greater Mecklenburg (CCPGM) [46] (hospitalizations, ED, clinic visits, medication compliance); local public sector data compiled by the UNC Charlotte Metropolitan Studies unit (http://mseap.uncc.edu/); and proprietary data purchased from Claritas, the Nielsen Company. An extensive range of geo-coded data will be accommodated in order to address the research issues. Specific variables will include levels of obesity, uncontrolled hypertension, housing density, neighborhood quality of life elements, recreational assets, grocery and food locations, street crime, pollution sources, public transportation systems, built environment elements, race/ethnicity, household income, public education data, including end of grade scores and absenteeism from Charlotte-Mecklenburg Schools; and, patient surveys and focus group findings (Table 1).

**Table 1:** Data elements for analysis of social determinants and health outcomes

	**Element**	**Source**	**Frequency**
Input/Independent Variables	Race/Ethnicity, Age, Gender	Claritas/UNCC Metro Studies, Hospital Billing Data	6-12 months
	Household Income	UNCC Metro Studies	12 months
	Housing Density	UNCC Metro Studies	12 months
	Distance to nearest grocery store	UNCC Metro Studies	12 months
	Distance to Nearest Park/Green Space	UNCC Metro Studies	12 months
	Sidewalk Access in Neighborhood	UNCC Metro Studies	12 months
	Distance to Public Transportation	UNCC Metro Studies	12 months
	Distance to Primary Care Clinic	UNCC Metro Studies	12 months
	Living within a Primary Care Clinic Service Area	CHS Billing Data/Geographic Retrofitting Model [44]	12 months
	Utilization of a Primary Care Clinic	CHS Billing Data	6 months
	Percentage of Free Lunches in Neighborhood School	UNCC Metro Studies	12 months
	Distance to Pollution Sources(Hazardous Materials, Coal Burning Plant)	UNCC Metro Studies	12 months
	Number of Food Stamps Users in Neighborhood	UNCC Metro Studies, Dept. of Social Services	12 months
	Social Network Analysis	Survey Data Collection	Ongoing
Outcomes Dependant Variables	All Cause Hospitalizations in past year (yes/no)	CHS Hospital Billing Data	6 months
	All Cause ED Visits in past year (yes/no)	CHS Hospital Billing Data	6 months
	ED Visits for Primary	CHS Hospital Billing	6 months
	Care Preventable or Treatable Illness in past year (yes/no)	Data/NYU Algorithm [62]	
	Utilization of a Primary Care Clinic in past year (yes/no)	CHS Hospital Billing Data	6 months
	School Absenteeism Over 6 days (yes/no)	UNCC Metro Studies/CMS School Data	12 months
	School Performance – passed EOG (yes/no)	UNCC Metro Studies/End of Grade Test Scores	12 months
	Perceived Quality of Life & Health Status (Aim 3,Validation)	Survey and Qualitative Data Collection	Ongoing

### Creation of geospatial models

**Figure 1:**
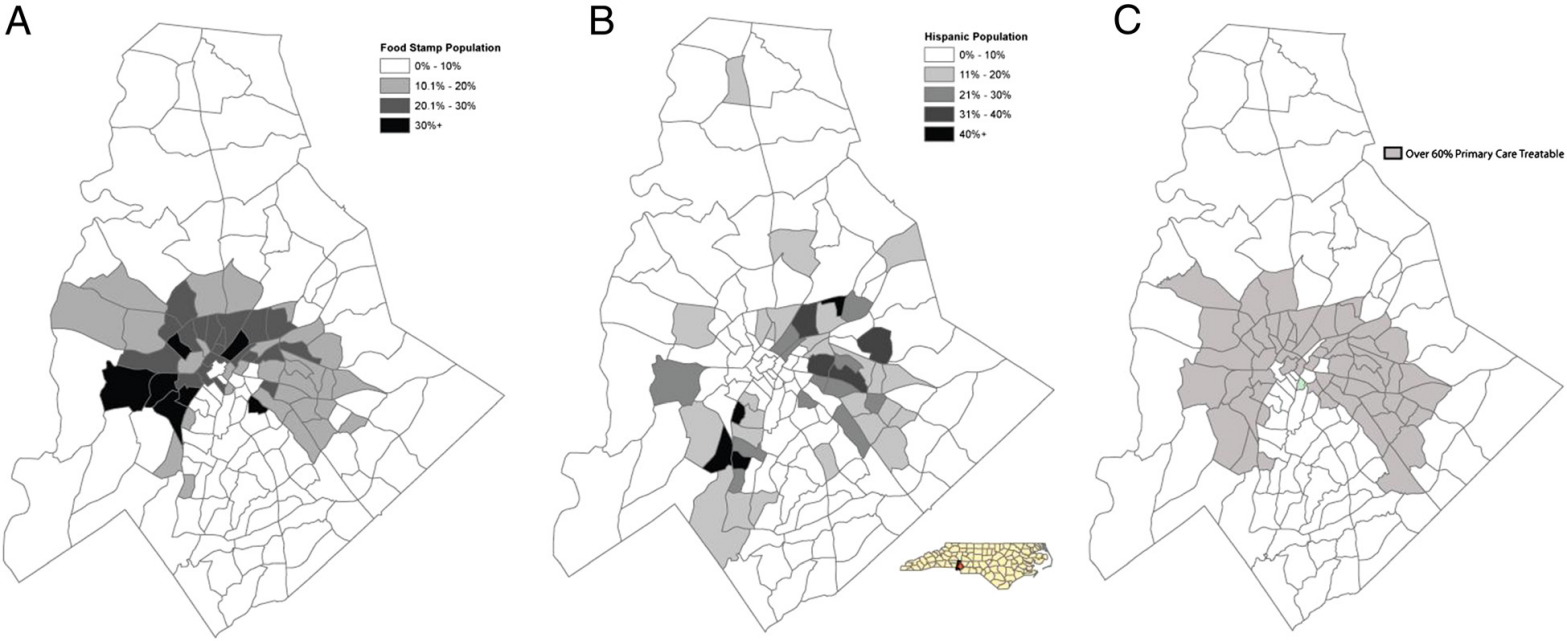
**Maps providing examples of individual variables mapped across Mecklenburg County, NC.** All data was collected in 2009. Map **A**. Data from the Mecklenburg County Department of Social Services was used to identify areas in the community with high percentages of food stamp utilization. Map **B**. Data from the UNCC Center for Metropolitan Studies and Claritas shows Hispanic Settlement across the county. Map **C**. Hospital data from the 5 county Emergency Departments analyzed with the NYU algorithm to identify areas in the community that are utilizing the ED for primary care treatable or preventable conditions (note shaded areas in Map C show census tracts where over 60% of all ED visits are primary care treatable or preventable). Maps like these will be created for all variables and shared with the advisory board to initiate the MultiAttribute Primary Care Targeting Strategy (MAPCATS) Process. (note small locator map of North Carolina).

For this project, a combination of GIS mapping tools will be utilized to create models showing past, current, and projected patterns of healthcare access at the neighborhood and city/countywide scales. These methods have previously been used by MAPPR on other projects (Figure 1) [47,48].

In earlier work, the MAPCATS model [47,48] relied solely on data describing the primary care safety-net and emergency department utilization in combination with citywide and neighborhood demographics. The newest modeling will be enhanced by incorporating additional variables available through the database (Table 1). To best determine the cumulative variable combination in blending population and healthcare indicators into informative maps, a mixed methods approach will be utilized. A three-step process will be carried out. First, all county address data will be geocoded using ArcGIS (ESRI, Redlands, CA). Prior work with this data has allowed us to achieve a 97% match rate. Second, neighborhood descriptive data including population density, household income, housing quality and density, crime rates, demographics, access to food, community walkability, and transportation options will be imported into ArcGIS. Finally, the CAB will review maps of each individual attribute (see sample maps in Figure 1) and assign weights based upon the perceived importance of each variable on health care access using a combination of the Analytic Hierarchy Process [49,50] and Multiple Attribute Assessment and Evaluation process [51].

One indicator of primary care need is the inappropriate use of the Emergency Department (ED) for primary care treatable or preventable conditions. The NYU Algorithm was developed as an indicator of the ability of the health delivery system to provide primary care services [52-54]. For this project, all ED data for Charlotte and Mecklenburg County will be updated and geocoded on a six-month cycle, the NYU algorithm will be used to sort the data, and results will be mapped and incorporated into the geospatial models. Descriptive summaries of all neighborhood variables will be developed. In turn, these localized, updated data will offer a better understanding of the relative distribution of the data and the populations being studied. Summaries will include race/ethnicity, age, and income level, using the poverty thresholds defined by the US. Department of Health and Human Services [14].

Following these steps, members of the research team and CAB will rank each of the selected attributes according to its perceived importance. The attributes will then be rated on a scale of 0 to 1, with 1 being the highest level of importance. The attributes will be combined and maps showing the data will be created and reviewed by the CAB and researchers for reliability [55-59]. Using this methodology, the perception and experience of the CAB is factored into assessments of the relative importance of each attribute in the model.

Following on, differences in ED usage between Hispanics and other ethnic groups will be tested by comparing the percentage of each subgroup with each outcome - hospitalization, emergency department visit, and emergency department visit for a primary care treatable or preventable condition.

#### Validation of geospatial models using qualitative methods and surveys

At strategic points throughout the research process, qualitative methodologies – specifically focus groups, key informant interviews and surveys with Hispanic community members, healthcare and social service providers will be conducted. The purposes for individual qualitative analyses are varied, but, in general, respond to the need to carefully move forward on behaviorally centered modeling. Specific concerns include validating the selection of variables for inclusion in the database; confirming that assumptions about data and model output reflect the lived experience and social networks; validating the geo-spatial model design and outputs; and reviewing the design and effective implementation of the intervention; and measuring its effectiveness in meeting goals of improved health care access for Hispanics in Mecklenburg County.

The focus group process will encompass four categories of participants: Hispanic community members; health care providers; combined community members and providers; and CAB members. In each case, focus groups will be comprised of 8 to 12 panelists and run for between 90 to 120 min. Discussion components of focus groups will be tape recorded and fully transcribed for analysis by research team members. Content analysis and thematic coding of the transcripts will be conducted with the assistance of ethnographic software such as NVIVO (Cambridge, MA). Guided by the preference and needs of participants, all of the community focus groups will be conducted in Spanish and translated into English for analysis. Focus groups will be held in easily accessible locations – including local churches, community centers and partnering health clinics. Before being submitted to the IRB, focus group discussion guides will be developed by the research team for CAB review, enhancement and approval. In total we aim to conduct 100 focus groups across the four different categories with approximately 20 focus groups being held in each of the five years of the project.

Each of the focus group categories will have a targeted subject area, based upon the participant’s role in Hispanic health care provision or client status. The following section presents these topics as well as the recruitment of participants and the frequency of meetings for each category. The Qualitative Analysis will include focus group discussions with:

1. The CAB will focus upon development of the database, selection and prioritization of the variables for inclusion in the model and assessment of the research process and intervention. This focus group will meet twice every year.

2. The health care provider focus group will target the appropriateness of the variables selected for the data base, geo-spatial model design, and output and idea generation about the design, implementation and effectiveness of the intervention. The provider focus group participants will be recruited through a snowball sampling approach that begins with providers from within the MAPPR network and then expands to ensure the broadest array of participants possible. The health care provider group will meet five times annually.

3. Community members will also concentrate on the validation of variable selection for data base, geo-spatial model design, output and idea generation about the design, implementation and effectiveness of the intervention. However, community discussions will also have an additional layer of strategic focus. In the early years of the project, community members will be drawn from across the county, with recruitment done in partnership with and through open advertising at the Latin American Coalition. But, in later years of the project, after specific neighborhoods have been identified as having health care deficiencies and interventions are needed, community participants will be recruited from these targeted neighborhoods. At that juncture, the focus group discussion will center around the validity of the variables in the model reflecting real world experiences; suggestions for the design and implementation of intervention strategies in the context of the neighborhood place; and ultimately, appropriate assessment of the intervention from a Hispanic community perspective. In this way, both the social and spatial networks and everyday lived realities of Hispanic neighborhood members will be reflected in both the research process and the intervention outcomes. The community member focus groups will meet most frequently, eight times per year.

4. A combined group of health care providers and community members will seek to achieve the same goals as groups two and three, while also providing an opportunity for productive dialogue between the two groups. In particular, health care providers can hear directly from community members their concerns and issues surrounding service access. Conversely, Hispanic community members can learn about challenges facing providers. The combined focus group will also offer a venue to build social networks within the structure of the project. The provider and community focus group will meet five times per year.

Two other qualitative methodologies will also be employed in this project. The first is a set of key informant interviews with a cross-section of five health care providers and five community representatives. The interviews will be held at the beginning and at the end of the project. The initial interviews provide an additional layer of depth and validation to the construction and modeling results. They will also permit the research team to probe further into the findings from the early focus groups. The end of project interviews offer summative discovery of the effectiveness of the intervention implementation and assessment of the project outcomes.

Finally, at the conclusion of the intervention stage of the project, a survey will be developed and administered to both, health care providers and Hispanic community members. This instrument will explore the experience and effectiveness of the intervention with a particular emphasis on the extent to which social and spatial networks have changed in the targeted neighborhoods over time and as a function of the CBPR process and intervention efforts. The survey will be based on validated surveys such as the Personal Resource Questionnaire 85 (PRQ-85 [60]). Additional questions will be added to the PQR85 to help better understand resources within a participant’s social network; access to healthcare services; use of informal health services; assimilation/acculturation; problem solving ability; and living in areas with a high density of Hispanic residents. This survey data will also be used to validate the geospatial models.

#### Design, implementation, and evaluation of a CBPR intervention to positively change the social networks of the target community in order to impact their utilization of primary care services

**Figure 2:**
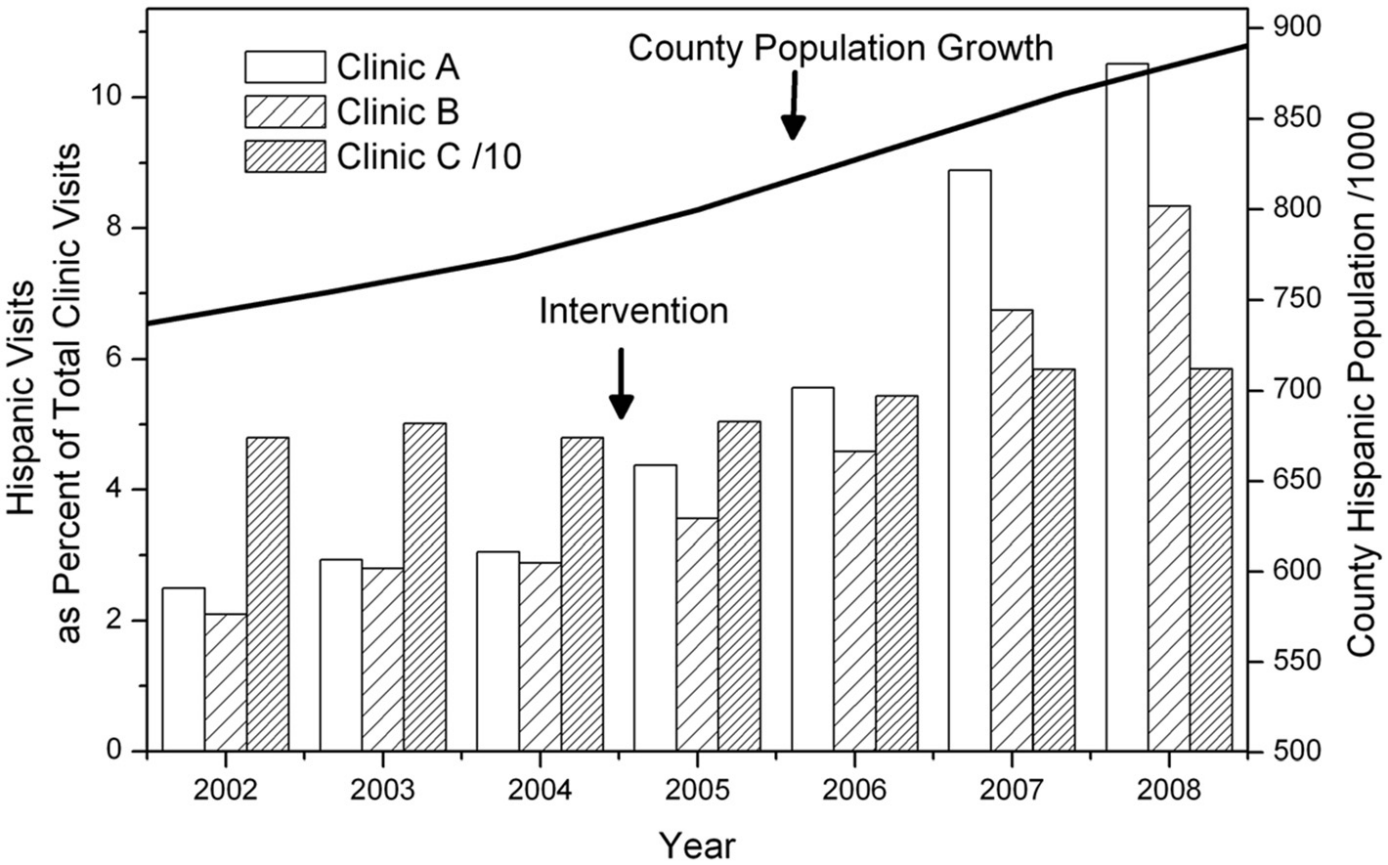
**Graph showing changes in clinic demographics for three community clinics.** Clinic A was part of a CBPR program assessing primary care needs for the Hispanic community between 2004 - 2008. No other intervention besides the use of the CBPR process occurred within Clinic A. During this time the clinic had over 180% increase in the number of Hispanic patients receiving care. One potential cause of this change was the partnerships and changes in the social networks of providers and community members created by the CBPR process itself.

Previous research by our network reveals changes in patterns of healthcare utilization that are associated with the use of CBPR. In particular, primary care clinics and providers that were involved in a preliminary CBPR project showed marked increases in the number of Hispanic patients seen in their clinics (Figure 2). The changes in utilization rates were not observed in similar clinics within the network that were not utilizing CBPR methods. In retrospect, it seems clear that the same process underlying CBPR (the creation of partnerships with community members and organizations) would also impact the social networks of the participants. These social networks appear to be of key importance for Hispanic newcomers and other vulnerable populations as they seek resources such as medical care [61].

The development of an intervention(s) will draw upon the key principles of CBPR (primarily developing equitable partnerships and community capacity) as a means in itself of improving access to primary care. The full intervention will be developed by the CAB. The CAB will prioritize guidelines for the intervention; and utilize the geospatial models developed to identify two neighborhoods to implement the intervention as well as two control neighborhoods. The research team will assist in the neighborhood selection process in order to ensure that the communities have similar population densities, socio-economic characteristics, proportions of Hispanic settlement, and resources. The intervention will be developed during the first year of this project and deployed 18 months after the project has started once the geospatial models have been completed and received initial qualitative validation. The research team will measure the social network of participants before and after the intervention using variations of validated measures of social network and social support (Medical Outcomes Study – Social Support Survey). This survey takes approximately 10 min to complete. It provides in-depth information about an individual’s social support network, and has been tested in both English and Spanish. In addition, the research team has developed a short questionnaire that allows us to collect additional information about the strength of a respondent’s social network, the presence of health providers within the network, and resources within the participant’s social network which have been shown to facilitate access to care. We will also collect information about the participants’ perceived health status using the SF-12 (Quality Metric, Lincoln, RI), as well as, recording chronic medical conditions and/or the use of medications on a regular basis.

The study design for the intervention will be developed by the collaborative team using CBPR techniques and closely informed by the geospatial model. All participants will be consented and asked to allow the research team to retrospectively and prospectively monitor their utilization of healthcare services. All visits to the outpatient and inpatient facilities of CHS will be reviewed starting two years prior to enrollment, and prospectively monitored from the time of enrollment through the end of the study (providing a total of four years of observations).

## Analysis

### Quantitative data analysis

First, descriptive summaries of all variables will be provided to the CAB by the research team in order to understand the relative distribution of the data and the populations being studied. Summaries will be based on race/ethnicity, age, and income level, centered around poverty thresholds defined by the US. Department of Health and Human Services [14]. Next, differences in outcomes for Hispanic residents compared to other subgroups will be tested by comparing the percentage of each subgroup with each outcome - hospitalization, emergency department visit, and emergency department visit for a primary care treatable or preventable condition (see NYU Description). We will use Kendall’s tau-c for each variable with significance occurring when p < 0.05.

Secondly, a multiple logistic regression analysis will be carried out. This technique will allow for an investigation of the simultaneous relationship of the continuous and categorical variables describing social determinants of health on the dichotomous outcome variables of all cause hospitalizations, all cause ED use, inappropriate ED use (as determined by the NYU algorithms, utilization of primary care services, and school-based outcomes of absenteeism and performance (Table 1). Note that primary care utilization will be considered an independent variable that may impact hospitalizations and ED use as well as a dependant variable that can vary based on other social determinants of health (e.g. income). Statistically significant correlations that are identified between the dependent and independent variables will be used to inform the development of the geospatial models.

### Survey analysis

The survey will be administered to 100 Hispanic members living in the two targeted neighborhoods and will be compared to 100 community members living in two paired neighborhood areas (n = 200). To accomplish this, 1600 surveys will be mailed to addresses randomly selected within each area. Prior MAPPR experience shows an approximate 25% response rate when surveys are mailed by a community partner. This sample will have sufficient power to detect a 3% difference in the PRQ-85 scores.

## Discussion

The results of this study will demonstrate the importance of several novel approaches to ameliorating health disparities within the Hispanic immigrant population including the use of CBPR, the effectiveness of community interventions that target social networks, and the importance of primary care access in ameliorating health disparities. Research-based, neighborhood scale interventions often experience difficulty in securing community acceptance and participation. To overcome these limitations, this study was designed to be developed and implemented using participatory methods that allow the intervention to be community-designed, led and assessed in partnership with the research team. Identifying new mechanisms that improve health outcomes is an important step towards advancing patient outcomes, avoiding preventable Emergency Department visits and hospitalizations, while simultaneously reducing overall healthcare costs.

## Competing interests

The authors declare that they have no competing interests.

## Authors’ contributions

All authors made significant contributions to the conception and design of this study and read and approved the final manuscript. MD, HS, OF, BUH and HT drafted the manuscript.

## Pre-publication history

The pre-publication history for this paper can be accessed here:

http://www.biomedcentral.com/1471-2458/12/769/prepub
